# Elongation factor-2 phosphorylation in dendrites and the regulation of dendritic mRNA translation in neurons

**DOI:** 10.3389/fncel.2014.00035

**Published:** 2014-02-10

**Authors:** Christopher Heise, Fabrizio Gardoni, Lorenza Culotta, Monica di Luca, Chiara Verpelli, Carlo Sala

**Affiliations:** ^1^CNR Institute of Neuroscience and Department of Biotechnology and Translational Medicine, University of MilanMilan, Italy; ^2^Department of Pharmacological and Biomolecular Sciences, University of MilanMilan, Italy; ^3^Neuromuscular Diseases and Neuroimmunology, Foundation Carlo Besta Neurological InstituteMilan, Italy

**Keywords:** eEF2, eEF2K, translation, neurons, dendrites, synapses, synaptic plasticity

## Abstract

Neuronal activity results in long lasting changes in synaptic structure and function by regulating mRNA translation in dendrites. These activity dependent events yield the synthesis of proteins known to be important for synaptic modifications and diverse forms of synaptic plasticity. Worthy of note, there is accumulating evidence that the eukaryotic Elongation Factor 2 Kinase (eEF2K)/eukaryotic Elongation Factor 2 (eEF2) pathway may be strongly involved in this process. Upon activation, eEF2K phosphorylates and thereby inhibits eEF2, resulting in a dramatic reduction of mRNA translation. eEF2K is activated by elevated levels of calcium and binding of Calmodulin (CaM), hence its alternative name calcium/CaM-dependent protein kinase III (CaMKIII). In dendrites, this process depends on glutamate signaling and N-methyl-D-aspartate receptor (NMDAR) activation. Interestingly, it has been shown that eEF2K can be activated in dendrites by metabotropic glutamate receptor (mGluR) 1/5 signaling, as well. Therefore, neuronal activity can induce local proteomic changes at the postsynapse by altering eEF2K activity. Well-established targets of eEF2K in dendrites include brain-derived neurotrophic factor (BDNF), activity-regulated cytoskeletal-associated protein (Arc), the alpha subunit of calcium/CaM-dependent protein kinase II (αCaMKII), and microtubule-associated protein 1B (MAP1B), all of which have well-known functions in different forms of synaptic plasticity. In this review we will give an overview of the involvement of the eEF2K/eEF2 pathway at dendrites in regulating the translation of dendritic mRNA in the context of altered NMDAR- and neuronal activity, and diverse forms of synaptic plasticity, such as metabotropic glutamate receptor-dependent-long-term depression (mGluR-LTD). For this, we draw on studies carried out both *in vitro* and *in vivo*.

## Introduction

The well conserved, ubiquitous eukaryotic Elongation Factor 2 kinase (eEF2K)/ eukaryotic Elongation Factor 2 (eEF2) pathway involves the phosphorylation and inactivation of eEF2 on Thr56 by eEF2K, thereby leading to an arrest of mRNA translation (Nairn et al., 1985; Mitsui et al., 1993; Ryazanov et al., 1997). Since eEF2K activity is regulated by calcium/CaM (Nairn et al., 1985; Mitsui et al., 1993), this pathway is of great interest to the field of neuroscience. Numerous papers have shown that dendritically localized eEF2K activity is altered by manipulating neuronal activity and glutamate signaling, owing to downstream events such as N-methyl-D-aspartate receptor (NMDAR) activation and subsequent increases in calcium levels (Marin et al., 1997; Scheetz et al., 2000; Lenz and Avruch, 2005; Maus et al., 2006; Sutton et al., 2007; Barrera et al., 2008; Autry et al., 2011), as well as metabotropic glutamate receptor (mGluR) activation (Park et al., 2008; Verpelli et al., 2010). Additionally, the eEF2K/eEF2 pathway is associated with diverse forms of synaptic plasticity (Chotiner et al., 2003; Kanhema et al., 2006; Davidkova and Carroll, 2007; Park et al., 2008; Seibt et al., 2012), most notably metabotropic glutamate receptor-dependent-long-term depression (mGluR-LTD), during which the pathway appears to regulate dendritic mRNA translation (Davidkova and Carroll, 2007; Park et al., 2008). Finally, while in general eEF2K activity and mRNA translation are negatively correlated in dendrites (Scheetz et al., 2000; Sutton et al., 2007), for not well understood reasons the translation rate of certain proteins like microtubule-associated protein 1B (MAP1B), alpha subunit of calcium/CaM-dependent protein kinase II (αCaMKII), and activity-regulated cytoskeletal-associated protein (Arc) actually increases when eEF2K activity is elevated in the context of altered neuronal activity and synaptic plasticity paradigms (Scheetz et al., 2000; Chotiner et al., 2003; Davidkova and Carroll, 2007; Park et al., 2008; Autry et al., 2011). Since these upregulated proteins have well-known functions at the synapse and in synaptic plasticity (Zalfa et al., 2003; Davidkova and Carroll, 2007; Park et al., 2008; Dajas-Bailador et al., 2012; Lisman et al., 2012; Wibrand et al., 2012) this raises the exciting possibility that the eEF2K/eEF2 pathway may regulate mRNA translation dendritically in a more complex manner than elsewhere, especially during activity-dependent synaptic changes. This may have the purpose of implementing the kind of local proteomic modifications that are necessary for plastic changes to take place at the postsynapse, a conceivable scenario, considering that dendrites harbor the components that are necessary for protein translation (Asaki et al., 2003; Swanger and Bassell, 2013).

## Manipulation of N-methyl-D-aspartate receptor (NMDAR) signaling and neuronal activity affects eukaryotic Elongation Factor 2 Kinase (eEF2K)/eukaryotic Elongation Factor 2 (eEF2) pathway-dependent mRNA translation in dendrites

There are several studies which address an NMDAR-dependent eEF2K/eEF2 pathway activation and subsequent dendritic changes in mRNA translation. The NMDAR is an obvious target for manipulating the eEF2K/eEF2 pathway at dendrites since it is located at the postsynapse, is permeable to calcium and is a crucial element of several signaling pathways (Collingridge et al., 2004; Prybylowski et al., 2005; Kessels and Malinow, 2009; Traynelis et al., 2010). Similarly, the eEF2K/eEF2 pathway at dendrites may be activated by increased neuronal activity since it involves glutamate signaling, which in turn leads to an influx of calcium via glutamate receptors (GluRs) such as the NMDAR and the α-amino-3-hydroxy-5-methyl-4-isoxazolepropionic acid receptor (AMPAR) (Malenka and Bear, 2004; Bear et al., 2007; Major et al., 2013). Additionally, alterations in neuronal activity and associated glutamate signaling could trigger other forms of dendritic eEF2K/eEF2 pathway activation, e.g., due to stimulation of mGluRs (Davidkova and Carroll, 2007; Park et al., 2008) which will also be addressed in this article.

In one study (Scheetz et al., 2000), 30 s pulses of glutamate and NMDA were used to stimulate NMDARs in synaptoneurosomes (fractions with enrichment of functional synaptic components) prepared from the rat superior colliculus. The authors found that even though total protein synthesis was reduced several minutes after the pulse, the translation rate of αCaMKII was actually increased. Importantly, NMDAR activation also led to an increase of eEF2 phosphorylation, strongly suggesting the involvement of the eEF2K/eEF2 pathway. They also demonstrated that using cycloheximide, a substance that blocks translation elongation independently of eEF2, lead to very similar proteomic changes. The authors therefore propose the following sequence of events: “NMDAR-mediated Ca^2+^ influx into dendrites activates Ca^2+^-dependent eEF2 kinase, which then phosphorylates eEF2. This phosphorylation might slow the local rate of protein translation, and elongation, rather than initiation, would consequently become the rate-limiting step in protein synthesis. Such a shift should favor upregulation of translation of abundant but poorly initiated transcripts such as αCaMKII in dendrites” (Scheetz et al., 2000). In another elegant study (Sutton et al., 2007), a microfluidic chamber was used which allows for the fluidic isolation of pre- and postsynaptic neurons. Application of tetrodotoxin (TTX) to the presynaptic compartment silenced presynaptic generation of action potentials while not interfering with miniature synaptic events/spontaneous neurotransmission. At the postsynaptic neuron a Green Fluorescent Protein (GFP) translation reporter was analyzed for 100 min after TTX application and interestingly an increase of eEF2 phosphorylation levels on Thr56 and a decrease of translation were reported as compared to baseline. Instead, applying TTX in addition with NMDAR blockers did not lead to this increase in phospho-eEF2 levels or decrease in translation, implying that NMDAR-mediated miniature excitatory synaptic events activate the eEF2K/eEF2 pathway and thereby lead to a decrease in translation. Additionally, using eEF2K inhibitors the authors were able to show that the decrease of translation that occurred during TTX treatment is due to activation of eEF2K, which is expected since eEF2K is the only known kinase regulating eEF2 (Nairn and Palfrey, 1987; Ryazanov et al., 1988; Mitsui et al., 1993; Dorovkov et al., 2002). The study concludes that the eEF2K/eEF2 pathway may act as a postsynaptic decoder of spontaneous and evoked neurotransmission (Sutton et al., 2007). A cautionary note, it is still unclear whether the available eEF2K inhibitors are well-suited to efficiently reduce eEF2 phosphorylation (Chen et al., 2011; Devkota et al., 2012), showing the need for the generation of new and efficient chemical compounds.

Two fascinating *in vivo* studies by Autry et al. (2011) and Nosyreva et al. (2013) used the NMDAR antagonist ketamine and eEF2K inhibitors to demonstrate that the eEF2K/eEF2 pathway regulates the expression of brain-derived neurotrophic factor (BDNF), a neurotrophin whose mRNA is found in dendrites (Tongiorgi et al., 1997, 2004; An et al., 2008) and is involved in numerous neuronal processes including synapse formation and synaptic plasticity (Reichardt, 2006). More specifically, they show that under resting conditions spontaneous glutamate release activates NMDARs which in turn engages eEF2K, resulting in the translational repression of BDNF. Consistently, acute administration of ketamine liberates BDNF expression and apparently alleviates depressive behavior in wildtype mice but not in eEF2K knockout mice, a fact that may prove to be useful in the context of major depressive disorder (Monteggia et al., 2013; Nosyreva et al., 2013). Importantly, the antidepressive effect appears to stem from BDNF-induced (presumably local) translation of AMPARs which become incorporated into the cell membrane and contribute to increased AMPAR-mediated synaptic transmission. In line with this fact, knockout mice for an AMPAR called GluA2 do not exhibit the antidepressive response induced by ketamin (Nosyreva et al., 2013). Interestingly, the finding that the (dendritically localized) eEF2K/eEF2 pathway leads to an activity-dependent upregulation of AMPAR currents also suggests that the activity of the eEF2K/eEF2 pathway may not only be dependent on network activity, but may itself determine the extent of network activity. Noteworthy, in opposition to the acute effect of ketamine, treatment with fluoxetine- another antidepressant- upregulates eEF2 phosphorylation in multiple brain regions only after chronic administration when antidepressive effects start taking place (Dagestad et al., 2006). This suggests that changes in eEF2K/eEF2 pathway-dependent mRNA translation enable not only acute but also chronic antidepressive effects, depending on the signaling cascade engaged by the antidepressant.

Most of the studies reviewed so far have implemented acute perturbation of NMDAR- and neuronal activity to look at eEF2K/eEF2-dependent changes of the dendritic proteome. Another interesting field of research revolves around the study of proteomic changes and associated events (such as changes in dendritic or spine morphology and synaptic transmission) which take place during prolonged modifications of network activity (Ehlers, 2003; Turrigiano and Nelson, 2004; Perez-Otano and Ehlers, 2005; Virmani et al., 2006; Turrigiano, 2008; Lazarevic et al., 2011). Two related studies (Piccoli et al., 2007; Verpelli et al., 2010) investigated the effect of prolonged changes in neuronal activity in primary neuronal cultures on the eEF2K/eEF2 pathway. The authors showed that increasing neuronal activity with bicuculline or lowering it with TTX for 48 h resulted in a dendritic increase of phosphorylation eEF2 on Thr56 or a decrease, respectively, strongly indicating an activation of eEF2K if neuronal networks are activated over longer periods of time (Verpelli et al., 2010). Verpelli et al. (2010) go on to show that activity dependent morphological changes of spine morphology depend on the presence of eEF2K, begging the question if there is a protein regulated by the eEF2K/eEF2 pathway that can account for the observed phenomenon. Indeed, the authors show that this protein is BDNF, whose mRNA translation is upregulated in dendrites in an eEF2K/eEF2 pathway-dependent fashion during long term bicuculline treatment. Interestingly, the bicuculline-induced increase of eEF2 phosphorylation and BDNF expression appears to depend on the activation of mGluRs rather than on the activation of AMPARs and NMDARs (Verpelli et al., 2010), suggesting that eEF2K activity can be modulated by a variety of GluRs.

Taken together, the data supports the notion that there are numerous ways of activating the dendritically localized eEF2K/eEF2-pathway, which can result from acute or prolonged stimulation of signaling elements like the NMDAR and mGluR (Nairn et al., 1985; Scheetz et al., 1997; Dorovkov et al., 2002; Chotiner et al., 2003; Davidkova and Carroll, 2007; Park et al., 2008; Verpelli et al., 2010; Autry et al., 2011; Tavares et al., 2012). Worthy of note, the proteomic changes induced by the activation eEF2K/eEF2 pathway can be quite diverse (and even opposite as in the case of BDNF and Arc), depending on which stimulation protocole is used. This may be due to the engagement of other signaling pathways but it may also mean that there is an extremely complex, protocole-specific eEF2K/eEF2 pathway-dependent change in the dendritic proteome which remains to be fully understood. For example, Im et al. (2009) show that the consolidation of fear memory involves an upregulation of BDNF and Arc synthesis in the hippocampus. However, hippocampal eEF2 phosphorylation is actually decreased during this process of memory consolidation, clearly contradicting the idea that a certain pool of proteins (like BDNF and Arc) is always positively correlated with an activation of the eEF2K/eEF2-pathway (Im et al., 2009). Lastly, a positive correlation between the activation of the eEF2K/eEF2 pathway and the translation rate of proteins may actually be unrelated in certain cases. For example, Panja et al. (2009) show that high frequency stimulation of neuronal populations leads to a phosphorylation of eEF2 and an increase Arc levels. However, the authors clearly show that in this experimental setting the activated eEF2K/eEF2 pathway is not responsible for the increase in Arc levels since pharmacologically blocking the pathway during the high frequency stimulation does not block the increase in Arc levels (Panja et al., 2009).

## Eukaryotic elongation factor 2 kinase (eEF2K)/eukaryotic elongation factor 2 (eEF2) pathway-dependent mRNA translation in dendrites in the context of synaptic plasticity

Synaptic plasticity refers to a “modification of the strength or efficacy of synaptic transmission” due to neuronal activity and has been discussed as the molecular correlate of phenomena like learning and memory (Citri and Malenka, 2008; Ebert and Greenberg, 2013). Two well studied forms of synaptic plasticity are long-term potentiation (LTP) and long-term depression (LTD), which increase or decrease synaptic transmission efficacy or strength, respectively, and whose maintenance apparently requires general and dendritic protein synthesis (Malenka and Bear, 2004; Citri and Malenka, 2008; Turrigiano, 2008). Since neuronal activity, synaptic plasticity, and mRNA translation are related events, the question arises whether the eEF2K/eEF2 pathway and synaptic plasticity are functionally related. Indeed, this appears to be the case for at least three well-established forms of synaptic plasticity, namely mGluR-LTD, chemically-, and BDNF-induced LTP (Chotiner et al., 2003; Kanhema et al., 2006; Davidkova and Carroll, 2007; Park et al., 2008).

Arguably, the most well understood relationship exists between the eEF2K/eEF2 pathway and mGluR-LTD. This form of synaptic plasticity can be electrically or chemically induced by mGluR agonists, is protein synthesis dependent, involves group I mGluRs (mGluR1 and mGluR5), and apparently also involves AMPAR-endocytosis (Citri and Malenka, 2008). In a fascinating study Park et al. (2008) pooled *in vitro* and *in vivo* data to show that under resting conditions, inactive eEF2K associates with group I mGluRs but can be liberated from the physical interaction with these receptors when they are stimulated by ligands. Active eEF2K then inhibits global translation at dendrites by phosphorylating eEF2. However, dendritic Arc mRNA translation is upregulated, which under resting conditions is usually suppressed by fragile X mental retardation protein (FMRP). Newly translated Arc then induces AMPAR-endocytosis, thereby completing the process of mGluR-LTD (Park et al., 2008). The authors’ notions are supported by their data showing that hippocampal slices of eEF2K-knockout mice do not exhibit mGluR-LTD, whereas previous work has shown that slices from FMRP-knockout mice exhibit exaggerated mGluR-LTD (Huber et al., 2002). Another study (Davidkova and Carroll, 2007), carried out in cultured neurons, also demonstrated that AMPAR-endocytosis following mGluR activation depends on the eEF2K/eEF2 pathway, since knocking down eEF2K abolishes this phenomenon. More specifically, after mGluR activation, eEF2K upregulates dendritic mRNA translation of MAP1B, which leads to the endocytosis of AMPAR, presumably because of an interaction of MAP1B and the AMPAR-associated protein Glutamate receptor-interacting protein 1.

The molecular basis of the relationship between the eEF2K/eEF2 pathway and LTP is less clear, even though the association visibly exists. It is also not clear whether this form of synaptic plasticity involves a dendritically or somatically located eEF2K/eEF2 pathway. The two forms of LTP that have been studied in this context are chemically-, and BDNF-induced LTP. Chemical LTP is induced by a combination of reagents (such as forskolin and tetraethylammonium) and depends on NMDARs, synaptic activity, cyclic adenosine monophosphate (cAMP)/adenylyl cyclase signaling, mRNA translation and gene expression (Chotiner et al., 2003; Zhao et al., 2012). BDNF-induced LTP also requires mRNA translation and gene expression but is induced by the infusion of BDNF (Kanhema et al., 2006). Interestingly, Chotiner et al. (2003) found that 1 h after induction of chemical LTP in the Cornu Ammonis area 1 of the mouse hippocampus protein synthesis was reduced but Arc mRNA translation was increased, reminiscent of the study of Scheetz et al. (Chotiner et al., 2003; Scheetz et al., 2000). As expected, eEF2 phosphorylation of Thr56 was increased which strongly indicates an activation of eEF2K in this experimental setting of chemical LTP. Since the increase of phosphorylation depended on cAMP/adenylyl cyclase activation and therefore engages protein kinase A (PKA) signaling (Voet et al., 2006), the authors hypothesize that eEF2K is activated during the induction of chemical LTP by PKA, which is known to phosphorylate and thereby activate eEF2K (Redpath and Proud, 1993a), resulting in the aforementioned changes in mRNA translation. Another study (Kanhema et al., 2006) addressed changes that are associated with BDNF-induced LTP. Among other things, the authors showed that inducing LTP by infusing BDNF into rat dentate gyrus lead to a transient phosphorylation of eEF2 on Thr56 in tissue homogenates, strongly suggesting an involvement of the eEF2K/eEF2 pathway in this form of synaptic plasticity. Worthy of note, the eEF2K/eEF2 pathway does not appear to be activated in dendrites during BDNF-induced LTP, but rather at non-synaptic sites, since the increase of eEF2 phosphorylation was not obtained when executing BDNF-induced LTP in synaptodendrosomes (fractions enriched in dendritic spine structures).

Importantly, the involvement of the eEF2K/eEF2 pathway is not limited to LTD and LTP, but instead has also been proven in the context of other forms of synaptic plasticity. One example of this is the participitation of the the eEF2K/eEF2 pathway in the context of monocular deprivation, which causes a reorganization of synapses and is a classical paradigm for inducing cortical synaptic plasticity. Seibt et al. (2012) showed that during sleep (when monocular deprivation-induced plasticity can occur) there is an increase of eEF2 phosphorylation and an increase in the translation of BDNF and Arc mRNA. These proteomic changes, which are presumably related to the activation of the eEF2K/eEF2 pathway, are necessary for synaptic plasticity to occur, indicating a strong involvement of the eEF2K/eEF2 pathway in the context of monocular deprivation-induced synaptic plasticity (Seibt et al., 2012). Curiously, eEF2 phosphorylation was observed in total lysates but not in synaptoneurosomes, indicating that in this specific experimental design for synaptic plasticity, the eEF2K/eEF2 pathway may not be engaged at the synapse but rather at the non-synaptic sites like in the soma. This result and the results of aforementioned work of Kanhema et al. (2006) suggest that not only the synaptically located eEF2K/eEF2 pathway, but also the somatically located eEF2K/eEF2 pathway may be important for synaptic plasticity to occur. In a further study, a connection between stress/sleep disruption were tied to the eEF2K/eEF2 pathway. More precisely, inducing stress by a combination of different factors like food deprivation, water deprivation etc., as well as sleep deprivation led to an upregulation of eEF2 phosphorylation levels in different parts of the brain, indicating a connection between the eEF2K/eEF2 pathway and these stressful events (Gronli et al., 2012). Since stress/sleep deprivation are known to impair plastic events at the synapse, this study highlights that there is, indeed, a strong and multifaceted link between the eEF2K/eEF2 pathway and synaptic plasticity, which is just beginning to be understood.

## New insights into the activation of the eukaryotic Elongation Factor 2 Kinase (eEF2K)/eukaryotic Elongation Factor 2 (eEF2) pathway and calcium-dependent eukaryotic Elongation Factor 2 Kinase (eEF2K) phosphorylation in neurons *in vitro*

**Figure 1:**
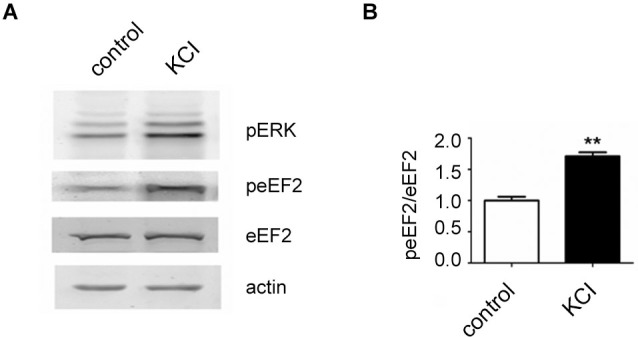
**Increase of eEF2 phosphorylation on Thr56 in neurons by KCL.** **(A)** Western blot analysis of neuronal lysates reveals a strong upregulation of phosphorylated ERK and eEF2 upon KCL treatment. Neuronal cultures were prepared from day 18 rat embryos (Charles River) and plated at medium density (200 cells/mm^2^) on 12-well plates and cultured with home-made B27 according to a protocole previously described (Romorini et al., 2004). At days *in vitro* 18, neurons were pretreated with TTX (1 μM) for 12 h and treated with KCL (55 mM) for 5 min. A strong increase of phosphorylated ERK on Thr202 and Tyr204 (pERK) and phosphorylated eEF2 on Thr56 (peEF2) can be appreciated. **(B)** Quantifications of normalized peEF2 band intensities (peEF2/eEF2) (vertical axis shows the mean fold change vs. control). Error bars are SEMs; ** *p* < 0.01 vs. control (student’s *t*-test).

In the course of this article several possibilities of activating the eEF2K/eEF2 pathway in neurons have been pointed out. We have found that a published protocol involving KCl, which leads to depolarization of neurons (Sala et al., 2000), can be utilized to increase eEF2 phosphorylation on Thr56 in primary neuronal cultures. For this, neurons were cultured as previously described (Romorini et al., 2004) and treated with KCl (55 mM) for 5 min at *days in vitro 18*. As expected, western blot analysis of lysates revealed a strong upregulation of phosphorylated extracellular signal-regulated kinase (ERK) on Thr202 and Tyr204 and, interestingly, a strong increase of phosphorylated eEF2 on Thr56 was also observed (Figures 1A, B). This is in line with the concept that depolarization of neurons and subsequent influx of calcium triggers eEF2K activation.

**Figure 2:**
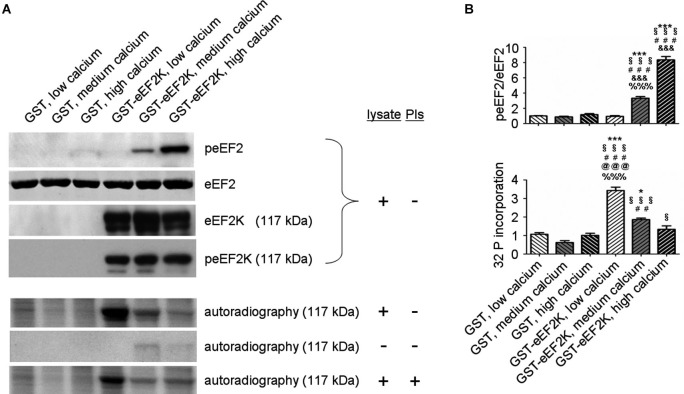
**eEF2K activity and total eEF2K phosphorylation are negatively correlated *in vitro.* (A)** Phosphorylation assays with varying levels of freely available calcium and subsequent western blot and autoradiographical analysis reveal a negative correlation between eEF2K activity and total eEF2K phosphorylation. Assays were carried in a total volume of 60 μl for 30 min at 37°C and had the following final composition: GST or GST–eEF2K preparations (3–5 μg), rat brain lysate (30 μg) or double distilled water (condition “- lysate”), HEPES (20 mM) pH 7.4, MgCl_2_ (10 mM), DTT (20 mM), ATP (100 μM; for subsequent western blot) or [γ-32P]ATP (100 μM; 5000 Ci/mmol; for subsequent autoradiography), and CaM (40 μg/ml). Depending on the group, ethylene glycol tetraacetic acid or CaCl_2_ (1 mM each) was added to mimic low calcium and high calcium levels, respectively. For medium calcium levels double distilled water was used to arrive at the total volume of 60 μl. For the condition “+ PIs” phosphatase inhibitors were added to the mix. For western blot analysis, the reactions were terminated by addition of sample buffer, whereas autoradiography was carried out on pelleted GST-eEF2K (centrifugation at 500 g for 1 min followed by addition of sample buffer). Western blot analysis (top) was done after phosphorylation assays containing lysates but no PIs. Immunodetection was carried out against peEF2, eEF2, eEF2K at 117 kDa (corresponding to molecular weight of GST-eEF2K, view Figure 3), and peEF2K (Ser 366; phosphorylation site of p70 S6K and p90 RSK) at 117 kDa. Autoradiography analysis (bottom) was done after phosphorylation assays a) with lysate but without PIs, b) without lysate or PIs, and c) with lysate and PIs. **(B)** Quantifications of normalized peEF2 (peEF2/eEF2) band intensities and autoradiographical band intensities (32 P incorporation) of assay (with lysate but without PIs) at 117 kDa (vertical axis shows the mean fold change vs. GST, low calcium). Error bars are SEMs; *, **, and *** *p* < 0.05, 0.01, and 0.001 vs. GST, low calcium; § vs. GST, medium calcium; # vs. GST, high calcium; & vs. GST-eEF2K, low calcium; @ vs. GST-eEF2K, medium calcium; % vs. GST-eEF2K, high calcium (ANOVA and *post hoc* Tukey test).

**Figure 3:**
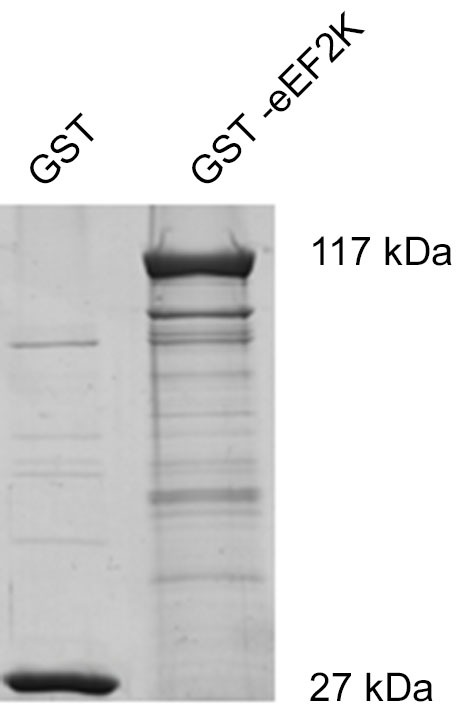
**Coomassie Brilliant Blue staining of GST-eEF2K reveals expected molecular weight of fusion protein**. eEF2K was expressed as the fusion protein GST-eEF2K (kind gift of Professor Chris G. Proud, University of Southampton) in BL21 competent *Escherichia coli* and purified as previously described (Tao et al., 2010; Pigott et al., 2012; Pyr Dit Ruys et al., 2012). After SDS-PAGE, gels were stained with Coomassie Brilliant Blue.

But what happens to the phosphorylation of eEF2K itself in neurons when it is activated by increasing levels of calcium? In this context, levels of eEF2K phosphorylation may be the result of calcium-dependent autophosphorylation (Mitsui et al., 1993; Redpath and Proud, 1993b; Pigott et al., 2012; Pyr Dit Ruys et al., 2012; Tavares et al., 2012), phosphorylation by another kinase, or dephosphorylation by a phosphatase. There are several identified upstream kinases of eEF2K such as PKA, p70S6 kinase (p70 S6K), and p90 ribosomal S6 kinase (p90 RSK) that regulate eEF2K phosphorylation in response to changes in cAMP-levels, ERK-signaling, and mammalian target of rapamycin-signaling, respectively (Redpath and Proud, 1993a; Wang et al., 2001; Browne et al., 2004; Browne and Proud, 2004; Lenz and Avruch, 2005). However, it has not been studied if the kinases upstream of eEF2K change their eEF2K-phosphorylation activity in response to increases in calcium levels though of course this is conceivable due to the broad effects of calcium signaling (Clapham, 2007; Chuderland and Seger, 2008; Fortin et al., 2013). To address the question of how eEF2K phosphorylation changes in response to elevated levels of calcium we bacterially overexpressed eEF2K as a fusion protein with glutathione S-transferase (GST) and purified it as previously described (Tao et al., 2010; Pigott et al., 2012; Pyr Dit Ruys et al., 2012). The resulting GST-eEF2K ran at about 117 kDa in sodium dodecyl sulfate-polyacrylamide gel electrophoresis (SDS-PAGE; Figure 3). The fusion protein was then used for a phosphorylation assay with rat brain lysates in which the availability of calcium ions was manipulated. After this, a western blot analysis of eEF2 phosphorylation or an autoradiographical analysis of GST-eEF2K phosphorylation was carried out as previously described (Gardoni et al., 2001) with minor modifications. As expected, eEF2 phosphorylation increased with rising calcium levels (Figure 2A, top; Figure 2B), which suggests an intact catalytic activity of the purified GST-eEF2K. Interestingly, GST-eEF2K phosphorylation was higher in low calcium than in high calcium (Figure 2A, bottom; Figure 2B) and this is probably not due to autophosphorylation since eEF2K autophosphorylates itself preferentially when calcium levels are increased (Mitsui et al., 1993; Redpath and Proud, 1993b; Tavares et al., 2012). Accordingly, carrying out the assay in the absence of lysate did not yield the aforementioned trend in GST-eEF2K phosphorylation (Figure 2A, bottom). Instead, carrying out the assay with the addition of phosphatase inhibitors (PIs) lead to a comparable trend in GST-eEF2K phosphorylation (Figure 2A, bottom). Altogether, this suggests that the higher phosphorylation of GST-eEF2K in low calcium is most likely due to upstream (possibly calcium dependent) kinases of eEF2K though we can exclude p70 S6K and p90 RSK since their phosphorylation site Ser366 (Wang et al., 2001; Browne and Proud, 2004) does not exhibit a calcium-dependent profile (Figure 2A, top). Possibly, the responsible kinases affect their phosphorylation sites on eEF2K in a calcium-dependent manner in order to regulate its activity. This would be an additional way to control the eEF2K/eEF2 pathway (and therefore mRNA translation) in response to changes of intracellular calcium levels which would imply an even more complex regulation of the eEF2K/eEF2 pathway than is already known.

## Conclusion

Phosphorylation of eEF2 via eEF2K in dendrites is one way for neurons to regulate dendritic mRNA translation. In general, activation of the eEF2K/eEF2 pathway leads to a dramatic reduction of mRNA translation. This also holds true for the subcellular compartment of dendrites, but interestingly the mRNA translation rate of a small subset of synaptic proteins with well-known synaptic functions is increased. Since eEF2K activity can be altered by the state of neuronal activation, this suggests the intriguing possibility that the eEF2K/eEF2 pathway may be utilized by neurons to implement proteomic changes at dendrites to facilitate activity-dependent plastic changes at the synapse.

## Author contributions

Christopher Heise wrote the text, planned and carried out the experiments; Fabrizio Gardoni corrected the text, provided expertise for the experiments, and carried out the autoradiography; Lorenza Culotta helped to carry out the experiments; Monica di Luca provided expertise and funding for the experiments; Chiara Verpelli corrected the text and helped in the organization of the GST-eEF2K DNA; Carlo Sala corrected the text, provided expertise and funding for the experiments.

## Conflict of interest statement

The authors declare that the research was conducted in the absence of any commercial or financial relationships that could be construed as a potential conflict of interest.

## Abbreviations

AMPAR: α-amino-3-hydroxy-5-methyl-4-isoxazolepropionic acid receptor
Arc: activity-regulated cytoskeletal-associated protein
BDNF: brain-derived neurotrophic factor
CaM: calmodulin
αCaMKII: alpha subunit of calcium/CaM-dependent protein kinase II
CaMKIII: calcium/CaM-dependent protein kinase III
cAMP: cyclic adenosine monophosphate
eEF2: eukaryotic Elongation Factor 2
eEF2K: eukaryotic Elongation Factor 2 Kinase
ERK: extracellular signal-regulated kinase
FMRP: fragile X mental retardation protein
GluR: glutamate receptor
LTD: long-term depression
LTP: long-term potentiation
MAP1B: microtubule-associated protein 1B
mGluR: metabotropic glutamate receptor
mGluR-LTD: metabotropic glutamate receptor-dependent-long-term depression
NMDAR: N-methyl-D-aspartate receptor
PI: phosphatase inhibitor
p70 S6K: p70S6 kinase
p90 RSK: p90 ribosomal S6 kinase
PKA: protein kinase A
SDS-PAGE: sodium dodecyl sulfate- polyacrylamide gel electrophoresis
TTX: tetrodotoxin
